# Hybrid Mix - a new software solution and web service to get surgical splints in orthognathic surgery

**DOI:** 10.1186/1746-160X-10-S1-O12

**Published:** 2014-12-12

**Authors:** Nicolas Nimeskern

**Affiliations:** 1 Clinique Diaconat-Fonderie, Mulhouse, France

## 

Planar cephalometry in essence provides only 2D information. CT scan provides 3D information for osseous and soft tissues but remains irradiant. STL dental models are interesting tools allowing for the production of 3D printed surgical splints.

In orthognathic surgery planning, exploiting these three fields of information by linking them to the clinical findings and getting them all synchronized is subject to imprecisions or even errors. The final aim remains to get a class I occlusion and to put the dental arches in the correct place in the facial architecture. All the surrounding osseous structures are not yet accessible to cutting guides and their osteotomies and synthesis remain therefore only dependent of the surgical technique of the practitioner.

Trying to simplify the approach, the authors present a new software which is and hybrid, enhanced, mixed, virtual articulator based on Delaire’s 2D cephalometry.

• Hybrid, because the classical clinical approach is transposed in a 3D virtual environment where the disadvantages of 3D are avoided.

• Enhanced, because allowing extruding 2D pertinent information from 2D documents in 3D where they become 2D information.

• Mixed, because allowing mixing 2D clinical pictures with 3D STL models.

• Virtual, because based on a virtual environment and on a linked web service.

• Articulator, because allowing to get the 3D printed splint.

This new approach is presented as well as the very beta version of the software.

